# Environmentally Just Futures: A Collection of Community-Driven African Environmental Education and Improvement Initiatives

**DOI:** 10.3390/ijerph19116622

**Published:** 2022-05-29

**Authors:** Onyemaechi Nwanaji-Enwerem, Andrea A. Baccarelli, Brian D. Curwin, Ami R. Zota, Jamaji C. Nwanaji-Enwerem

**Affiliations:** 1 Department of Family Medicine and Community Health, Duke University School of Medicine, Durham, NC 27710, USA; on18@duke.edu; 2 Department of Environmental Health Sciences, Mailman School of Public Health, Columbia University, New York, NY 10032, USA; ab4303@cumc.columbia.edu; 3 Division of Field Studies and Engineering, National Institute for Occupational Safety and Health, Centers for Disease Control and Prevention, Cincinnati, OH 45226, USA; bic4@cdc.gov; 4 Department of Environmental and Occupational Health, The George Washington University Milken Institute School of Public Health, Washington, DC 20052, USA; azota@gwu.edu; 5 Gangarosa Department of Environmental Health, Emory Rollins School of Public Health, Atlanta, GA 30322, USA; 6 Department of Emergency Medicine, Emory University School of Medicine, Atlanta, GA 30322, USA; 7 Division of Environmental Health Sciences, School of Public Health, Center for Computational Biology, University of California, Berkeley, CA 94720, USA

**Keywords:** environmental health, recycling, reforestation, SDG, solar, exposure science, environmental justice

## Abstract

Advocating for healthy environments is a matter of justice. Changes in environments have tremendous impacts on the health of communities, and oftentimes, individuals are unable to safeguard themselves through individual actions alone. Efforts frequently require collective action and are often most effective when led by the communities most impacted. In this spirit, we launched *“Vibrations”*, an African environment photo essay contest. Through funding and publicity, we aimed to support community-led environmental improvement and education initiatives presently taking place on the continent. We received nearly two dozen submissions and selected eight winners. The winners come from five countries (Ghana, Kenya, Mozambique, Nigeria, and South Africa) and have taken on a range of projects aimed at improving environments across a variety of African regions. Projects included efforts to combat pollution, create environmentally conscious school curricula, utilize clean energy sources, and spread awareness about environmental justice concerns in local communities. It is our hope that this report highlights these transformative community-driven efforts, promotes continued conversations on environmental justice in Africa, and encourages meaningful action via policy changes and collaborations throughout the African continent and beyond.

## 1. Introduction

When approached by the British Broadcasting Company (BBC) journalist, a young Nigerian woman responded, “Everything is now hot because we cut down so many trees in our environment. Like my house now, we don’t have any trees again and we use to have [them] before”. Another young woman from Kenya offered a similar sentiment when asked what the climate movement meant to her: “It means we have to fight for our environment and voice out what we want…Today, I want the government to listen to its people and do what is right for mother nature…stop this coal mining…take care of our environment…and restore our forests [1]”. Although environmental justice (EJ) as a discourse has its roots in the United States [2], these BBC perspectives reflect the continued need for EJ frameworks and practices all over the globe, especially in Africa. Africa is responsible for approximately 4% of global carbon emissions [3] but bears a disproportionate burden of climate change impacts, including desertification, deforestation, rising sea levels, droughts, and declining agricultural productivity [4,5]. The continent is further burdened by ongoing environmental injustices such as commercially related oil spills that have for decades polluted the air and water of individuals living in communities such as the Niger Delta region of Nigeria [6]. Moreover, rapid urbanization in areas such as Kinshasa, Democratic Republic of Congo have resulted in the urban poor bearing a disproportionate burden of the challenges with solid waste management [7]. Hence, although Africa is poised to continue unprecedented levels of economic and social growth, EJ principles must be centered to avoid repeating harms of the past and to minimize the creation of future harms. 

Given consideration to the literature that recommends engaging and following the lead of communities as an effective approach for achieving EJ goals [8,9,10], in the summer of 2021, we sent out a call via social media platforms to launch *“Vibrations”*, an African environment photo essay contest. The contest had two major aims. First, we wanted to draw greater attention to ongoing environmental improvement and education efforts on the African continent. As such, full photo essays will be made publicly available online. Secondly, we wanted to provide funding to project leaders to help continue their efforts. Participants were asked to submit essays outlining the rationale for their environmental projects and photos demonstrating progress made and ongoing efforts. Submissions were judged by a committee of environmental and public health experts on the criteria of the impact/scale of the work, community buy-in, and project sustainability. After reviewing submissions from across the continent, we selected eight winners whose transformative projects we highlight below.

## 2. Projects

**Figure 1:**
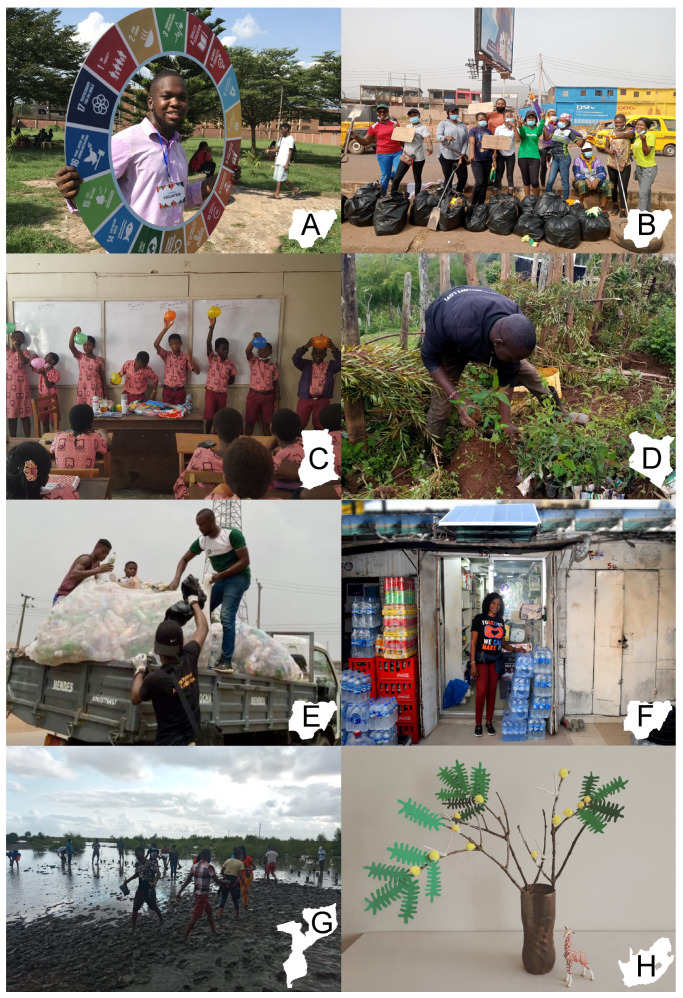
**Environmental education and improvement initiatives.** Depicted are the community-driven environmental education and improvement initiatives led by photo essay contest winners. Winners arose from five different countries (Ghana, Kenya, Mozambique, Nigeria, and South Africa). Projects included sustainable development goal awareness campaigns (**A**), clean-up initiatives (**B**), grade-school education programs (**C**), reforestation projects (**D**,**G**), recycling campaigns (**E**), renewable energy installation projects (**F**), and the creation of eco-art (**H**). Authors were granted permission to use and publish the photographs.

1. **Sustainable Developmental Goals (SDGs) Walk Campaign 2021** (Oluwaseun Adebayo, Edo, Nigeria): This event took place on 8 October 2021, in Benin City. Through a series of activities including environmental cleanups and information booths, the event aimed to increase local community awareness of the 17 United Nations (UN) SDGs (Figure 1A).
2. **Cash for Trash** (Ogechi Nwonye, Enugu, Nigeria): This ongoing project aims to simultaneously address the issues of poverty and environmental improvement. Currently, the project team is working with schools and other community organizations to develop programs where recyclables from rural and semi-urban areas can be exchanged for school fees, food items, or cash for low-income families. The project also hopes to improve climate literacy throughout the community (Figure 1B).
3. **Recycle Up!** (Salim Abubakar, Kumasi, Ghana): This community-based waste recycling project believes that plastic waste can be effectively decreased through grassroots behavior change. Through initiatives, including recycling clubs in primary schools, the team is working to create a robust circular economy where all plastic wastes are effectively managed to prevent environmental pollution and generate resources (Figure 1C).
4. **Kapsowar Tree Planting** (Cornelius Kemboi, Kapsowar, Kenya): This month-long project aimed to increase tree cover in a local community while promoting reforestation and tree cultivation. In addition to planting, there was also an emphasis on fostering an awareness of the ecologic and economic importance of trees among volunteers and community members (Figure 1D).
5. **Project Save 1M Plastic Bottles** (Martin Okorowu, Imo, Nigeria): This ongoing green outdoor activity seeks to recover and recycle over one million plastic bottles, thereby diverting them from drainage systems, bodies of water, and dumpsites. The program also has the goal of engaging and educating youth to inspire positive waste management behaviors in Owerri (Figure 1E).
6. **The Love and Light Project** (Isaac Omoyele, Lagos, Nigeria): This project recognizes the losses in productivity from deficits in national grid electricity. The team views solar energy as a valuable and worthy solution to this and other social, economic, and environmental challenges. Hence, they aim to educate communities on clean energy and garner funding to replace fuel generators with solar kits (Figure 1F).
7. **Combating Oceanic Pollution and Mangrove Restoration** (Ivaldo Fumo, Quelimane City, Mozambique): This project has three parts. The first two areas focus on planting mangrove seedlings to reforest degraded areas and decontaminating existing mangrove areas soiled by plastic bottle waste. The final focus of the project is to educate and sensitize communities to develop better waste management habits (Figure 1G).
8. **Youth Earth Protector** (Romario Valentine, Durban, South Africa and Segera, Kenya): This young environmentalist works with community partners to plant as many trees as possible around Africa to help combat desertification, create sustainable jobs for the youth, provide food and shelter for wildlife, and slow climate change. He also creates eco-art from recyclable waste products to raise awareness about environmental issues (Figure 1H).

## 3. Reflections and Future Work

We asked the contest winners what efforts, if taken by additional stakeholders, would be most helpful for supporting their work. They offered a broad range of advice. They first suggested that all initiatives should include all age ranges when possible. Including children was particularly highlighted as important, as future generations are most likely to bear the burden of environmental decisions made today. Additionally, they called for more public and private grant opportunities. Not only do grants provide financial capital for initiatives, but they also provide opportunities to build lasting partnerships between organizations and communities that may be aligned in values. There was also a call to make information about environmental issues more publicly available. In addition to making research publicly available, radio jingles, movies, and talk shows could be leveraged as creative ways to highlight environmental issues so that others can read, learn, and participate. With specific attention to policy goals, some winners asked that solutions that have been shown to have health benefits (green energies, indoor air filters, etc.) be subsidized by local and federal governments to increase their adoption by the public.

One of our ongoing goals is to help translate community-based work to sustainable political action. We truly believe that if community programs can gather adequate momentum, they can influence broader policy changes. Nevertheless, such action often requires multiple stakeholders with diversified interests. Often, stakeholders go beyond any single region or country. One unique element of our contest that addresses this complexity is our international support. With recognition and funding from the International Society of Exposure Science and the International Society for Environmental Epidemiology, we are well-positioned to facilitate some of these cross-sector stakeholder relationships. Both societies have active members from academia, government, and the private sector. Moreover, both societies are constantly exploring mechanisms for increasing their impact and service on the African continent. In line with frameworks for social change [11], these mechanisms include, but are not limited to:

1. Framing EJ as a sustainable opportunity for employment and entrepreneurship.
2. Improving EJ education efforts for both governmental entities and the general public.
3. Growing research investments aimed at understanding and identifying community-specific EJ challenges.
4. Expanding community-based program investments that can adequately address identified challenges.

It is particularly important to emphasize that our future work will focus on more targeted efforts that promote EJ advocacy on the continent. Despite analytics demonstrating contest page views from all regions of the African continent, we did not receive any submissions from North and Central Africa. We are currently working on strengthening relationships with public and private organizations in these regions to remedy this issue for future efforts. The solutions to environmental health problems are as diverse as the environments and the people themselves. Although we do showcase an abundance of diversity through our *“Vibrations”* winners, it is our priority to continue using our resources to strengthen community-based efforts in even more diverse regions.

## 4. Conclusions

In conclusion, from desertification to groundwater contamination by oil spills and multi-toxin air pollution, Africa faces a considerable and unique set of environmental health challenges. Despite these hurdles, individuals and communities continue to push for changed behaviors and policies to help actualize a healthier future for the continent’s inhabitants. Ultimately, it is our sincere hope that featuring this and similar work spurs further support as well as public and private partnerships that continue to make EJ a priority on the African continent.

## Data Availability

Not applicable.
